# Pure Epigallocatechin-3-gallate-Assisted Green Synthesis of Highly Stable Titanium Dioxide Nanoparticles

**DOI:** 10.3390/ma17020275

**Published:** 2024-01-05

**Authors:** Bogdan Andrei Miu, Miruna Silvia Stan, Maria Mernea, Anca Dinischiotu, Ionela Cristina Voinea

**Affiliations:** 1 Department of Biochemistry and Molecular Biology, Faculty of Biology, University of Bucharest, 91–95 Splaiul Independentei, 050095 Bucharest, Romania; miu.bogdan-andrei@s.bio.unibuc.ro (B.A.M.); anca.dinischiotu@bio.unibuc.ro (A.D.); ionela-cristina.voinea@bio.unibuc.ro (I.C.V.); 2 Sp@rte Team, Institute of Genetics and Development of Rennes, UMR6290 CNRS, University of Rennes 1, 35042 Rennes, France; 3 Research Institute of the University of Bucharest (ICUB), University of Bucharest, 050657 Bucharest, Romania; 4 Department of Anatomy, Animal Physiology and Biophysics, Faculty of Biology, University of Bucharest, 91–95 Splaiul Independentei, 050095 Bucharest, Romania; maria.mernea@bio.unibuc.ro

**Keywords:** titanium dioxide nanoparticles, green synthesis, green approach, epigallocatechin gallate, titanium isopropoxide hydrolysis

## Abstract

Nanoparticles (NPs) are conventionally produced by using physical and chemical methods that are no longer in alignment with current society’s demand for a low environmental impact. Accordingly, green synthesis approaches are considered a potential alternative due to the plant extracts that substitute some of the hazardous reagents. The general mechanism is based on the reducing power of natural products that allows the formation of NPs from a precursor solution. In this context, our study proposes a simple, innovative, and reproducible green approach for the synthesis of titanium dioxide (TiO_2_ NPs) that uses, for the first time, the major component of green tea (*Camellia sinensis*)—epigallocatechin-3-gallate (EGCG), a non-toxic, dietary, accessible, and bioactive molecule. The influence of EGCG on the formation of TiO_2_ NPs was analyzed by comparing the physicochemical characteristics of green synthesized NPs with the chemically obtained ones. The synthesis of bare TiO_2_ NPs was performed by hydrolysis of titanium isopropoxide in distilled water, and green TiO_2_ NPs were obtained in the same conditions, but in the presence of a 1 mM EGCG aqueous solution. The formation of TiO_2_ NPs was confirmed by UV-VIS and FTIR spectroscopy. SEM micrographs showed spherical particles with relatively low diameters. Our findings also revealed that green synthesized NPs were more stable in colloids than the chemically synthesized ones. However, the phytocompound negatively influenced the formation of a crystalline structure in the green synthesized TiO_2_ NPs. Furthermore, the synthesis of EGCG–TiO_2_ NPs could become a versatile choice for applications extending beyond photocatalysis, including promising prospects in the biomedical field.

## 1. Introduction

Various industries have been impacted by the imperative worldwide demand for an environmentally friendly and more sustainable economy that has characterized the last decades. The urgency for embracing sustainable practices stems from the far-reaching consequences of climate change, loss of biodiversity, depletion of vital resources, and the health risks associated with the spread of environmental pollutants.

In the light of actual climate neutral initiatives, green nanotechnology has emerged as a promising research area that might avoid the risks associated with the conventional one. Different chemical and physical methods for nanoparticles (NPs) production have been developed, but these have been high cost and energy consuming, used toxic reagents and generated important quantities of waste products [1] with possible environmental consequences. The concept of green chemistry might offer several advantages, including increased energy efficiency and a minimized generation of hazardous waste and emissions compared to the actual branch of nanotechnology that deals with NPs’ production [2].

Among different types of NPs, titanium dioxide (TiO_2_) ones were of particular interest, being ordinarily used in sunscreen products for skincare [3]. Considering their photocatalytic properties, the range of applications of TiO_2_ NPs have been widened, including the development of equipment for wastewater and indoor polluted air purification [4], self-cleaning textiles and other surfaces [5], paints with a long-lasting antimicrobial effect [6], or enhanced food packaging [7]. It is not surprising that the production of TiO_2_ has increased over time [8].

The term ‘green’ in the aforementioned syntagma originally referred to the use of living plants. The green or biological synthesis of NPs was even observed as a natural phenomenon occurring in plants harvested from metal contaminated soils. These plants, which were exploited in phytomining, could hyperaccumulate and deposit metals in the form of NPs [9]. As the research progressed, other organisms including bacteria [10] and fungi [11], but also whole plant extracts and purified phytocompounds [12] were used in the NPs’ synthesis, extending the horizon of the green approach. These alternative sources have provided a wider range of accessible and low-cost options to produce NPs, allowing for greater diversity in their properties and potential applications. In consequence, contributions to the standardization of this approach and its scaling at an industrial level have been constantly made.

Regarding the synthesis of TiO_2_ NPs through green synthesis, the possible plant extracts include *Cicer arietinum* L., *Aloe vera*, *Annona squamosa*, *Catharanthus roseus*, *Cinnamomum tamala*, and others, as these were described in a recent review [13]. In addition, *Camellia sinensis*, the green tea plant, an accessible, cheap, and dietary source of biomolecules with medicinal benefits due to the abundance of phenolic compounds named catechins, could be a valuable choice for green synthesis. Epicatechin, epigallocatechin, epicatechin-3-gallate, and epigallocatechin-3-gallate (EGCG) are the main catechins in *C. sinensis*, the last one being the most prevalent (~60%) and also the most studied for its bioactivity [14]. The main advantage of EGCG is its antioxidant activity [15], but different studies also highlighted its antimicrobial [16] and anti-inflammatory effects [17]. Therefore, EGCG might be involved in cardiovascular protection [18] and prevention of cancer [19].

*C. sinensis* leaf extract was used for the green synthesis of gold [20], silver [21,22], palladium [23], selenium [24], magnesium oxide [25], copper oxide [26], iron oxide [27], as well as zinc oxide [28] NPs. To the best of our knowledge, the use of its components is not so extended, pure EGCG being involved so far in the green synthesis of gold [29], silver [30], and zinc oxide [31] NPs, respectively.

The mechanism by which metallic NPs are synthesized through green approaches is explained by the reduction power of phytocompounds. Briefly, the reduction of metallic ions within a precursor solution is driven by the transfer of electrons from biomolecules to these and leads to the formation of ordered structures. These provide a support for the growth of particles determined by further addition of the reduced ions [32]. The particles’ enlargement could be stopped by the same biomolecules that act also as capping agents [33].

However, due to the lack of an explanation for the conversion to oxides of the zero-valent state acquired by the element after bio-reduction, this theoretical model might not correspond to the synthesis of metallic oxide NPs. TiO_2_ represents a distinct case, as its synthesis could be obtained simply through hydrolysis of titanium alkoxides followed by condensation [34]. Alkoxides polymerize and form a 3D structure. When adding biomolecules in reaction, these might be trapped into the structure, changing the properties of NPs.

Our study describes for the first time the synthesis of TiO_2_ NPs based on the hydrolysis of titanium isopropoxide in the presence of pure EGCG. The novelty of this approach involves the fact that pure forms of phytochemicals are not well represented in research dealing with NPs’ synthesis, although they could bring useful information to the field. Because we identified a large knowledge gap regarding the modulation of the characteristics of green NPs by the biomolecules used for their synthesis, which could contribute to the impediment of scaling up green approaches at an industrial level, our research focused on the comparison of physicochemical properties of TiO_2_ NPs obtained by a classical chemical method and green synthesis in the presence of aqueous solution of EGCG. Therefore, our single-molecule approach could support the reliability of research in green NPs synthesis as we consider the acquired characteristics of green NPs are difficult to reproduce when using whole plant extracts due to their variability.

## 2. Materials and Methods

### 2.1. Chemical Synthesis of TiO_2_ NPs

Chemical TiO_2_ NPs (chem-TiO_2_ NPs) were synthesized by hydrolysis and condensation reactions starting from titanium (IV) isopropoxide (TTIP; conc. 97%, Sigma-Aldrich, St. Louis, MO, USA) as a precursor. The method was based on the previous work of Mahshid et al. [35] with certain adjustments. Compared to their approach, the pH of distilled water was not changed, additional washes of the obtained powder were introduced, and the annealing process was not performed.

A volume of 2 mL of TTIP was diluted in 6 mL of isopropanol. This solution was added dropwise into a beaker containing 100 mL of distilled water while being stirred at 400 rpm and heated to 60–70 °C. A white precipitate could be observed instantly. The mixture was left on the heating plate overnight (about 18–20 h) at 60–70 °C and under stirring to allow all the water to evaporate. This relatively moderate temperature of 60–70 °C leads to the slow evaporation of water while allowing condensation to take place (see Section 4). Higher temperatures might not be safe as TTIP is a flammable substance.

The obtained white powder was then washed once with ethanol and several times with distilled water by centrifugation at 5000 rpm for 20 min. The sediment was placed in a porcelain mortar and dried in an oven for 2 h at 100 °C. The resulting powder was dispersed using a pestle, sealed, and stored at 4 °C.

### 2.2. Green Synthesis of TiO_2_ NPs

For the green synthesis of TiO_2_ NPs (EGCG–TiO_2_ NPs), the volume of 100 mL of distilled water was replaced with 100 mL of 1 mM aqueous solution of EGCG (ab120716; Abcam, Cambridge, UK). The same procedure detailed in Section 2.1 was applied, but the precipitate formed had a brown color.

### 2.3. Physicochemical Characterization of TiO_2_ NPs

The synthesized NPs were further characterized using UV-VIS spectroscopy, Fourier-transform infrared spectroscopy (FTIR), scanning electron microscopy (SEM), X-ray diffraction (XRD), and dynamic light scattering (DLS). The UV-VIS spectra of colloidal solutions of TiO_2_ NPs were recorded from 200 nm to 700 nm by using a Jasco V550 UV-VIS spectrophotometer (Jasco, Inc., Tokyo, Japan).

The chemical composition of TiO_2_ NPs and possible biomolecules on the surface of the green synthesized ones were analyzed by FTIR using a Bruker Tensor 27 spectrometer (Bruker Optik GmbH, Ettlingen, Germany). The addressed frequency range was from 4000 cm^−1^ to 400 cm^−1^ with a resolution of 2 cm^−1^. Measurements were performed in ATR (attenuated total reflection) setup. The advanced ATR correction of data was performed using OPUS software (version 7.2, Bruker Optik GmbH, Ettlingen, Germany) in order to assure the compatibility of our results with transmittance measurements.

The size and morphology of NPs’ powders were analyzed through electronic microscopy. Micrographs were acquired using a scanning electron microscope from FEI Company (Eindhoven, The Netherlands) equipped with a standard Everhart–Thornley detector. Investigations were carried out in a high vacuum, with 30 kV acceleration voltage, a working distance between 9.5 and 9.7 mm, spot size set up at 3.5 nm, and a variable horizontal field width from 1.49 to 298 μm. The most representative micrographs were presented.

The XRD pattern of NPs’ powders were recorded using a PANalytical Empyrean diffractometer (PANalytical, Almelo, The Netherlands) by CuKα radiation (λ = 1.54056 Å) at room temperature and Bragg diffraction angles 2θ from 10 to 80°. XRD scanning was continuously performed with a step size set up at 0.02° and 20 s/step acquisition time, at 45 kV and 40 mA. The Rietveld method was used to refine the structure of chem-TiO_2_ sample.

DLS was performed to determine the size of NPs in colloidal aqueous solutions and their zeta potential by using a Nano ZS ZEN3600 Zetasizer (Malvern Instruments, Malvern, UK). All NPs dispersions were prepared at a concentration of 50 μg/mL in ultrapure water. Three measurements were performed for each sample at 25 °C using the refractive index of 1.47 for material and of 1.33 for solvent (water) to determine the particles’ size and zeta potential.

## 3. Results

### 3.1. Optical Properties of Green Synthesised TiO_2_ NPs

**Figure 1:**
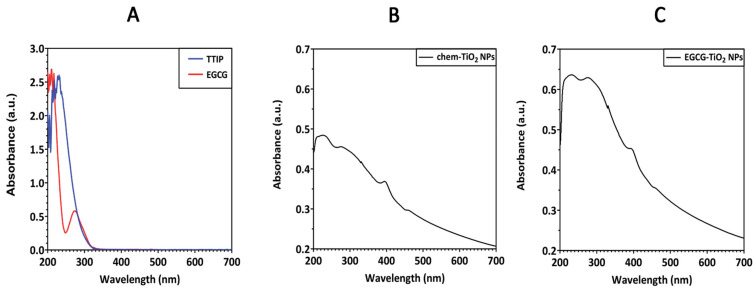
UV-VIS absorbance spectra of (**A**) TTIP and EGCG precursors, (**B**) chem-TiO_2_ NPs and (**C**) EGCG–TiO_2_ NPs.

UV-VIS spectroscopic analysis was performed to evaluate the formation of TiO_2_ NPs and to predict their photocatalytic activity. Considering that the spectra of purified TiO_2_ NPs have a different pattern than the precursors’ ones (Figure 1A), the spectroscopic data offered a preliminary proof that TiO_2_ particles were successfully formed in the designed reactions. Figure 1B,C depicts the UV-VIS spectra of TiO_2_ particles synthesized by hydrolysis of TTIP in distilled water and EGCG solution, respectively. The absorption spectrum of TiO_2_ NPs usually depends on several factors, including particle size, shape, and particle–particle interaction (agglomeration) with the medium. TiO_2_ absorbs light at wavelengths between 275 and 405 nm. In this case, the absorption maximum (λ_max_) for chem-TiO_2_ NPs was found at 226 nm while for the green synthesized EGCG–TiO_2_ NPs maximum absorption occurred at 232 nm. Both samples had an additional peak slightly below 280 nm, specifically 276 nm for chem-TiO_2_ NPs and 278 nm for EGCG–TiO_2_ NPs. Another peak at 395 nm could be observed in chem-TiO_2_ NPs’ spectra and it was blue-shifted in the case of EGCG–TiO_2_ NPs (392 nm). The sharp absorbance peak around 385–400 nm region confirms the formation of TiO_2_ NPs, indicating the charge coordinated electronic transition between the O (2p state) and Ti at 3d state [36].

**Figure 2:**
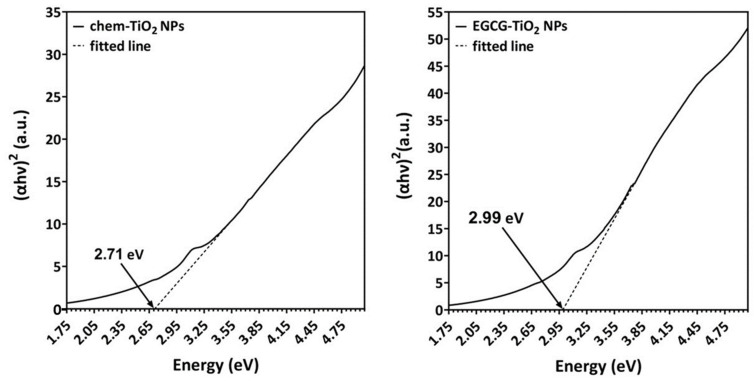
Tauc plot of chem-TiO_2_ NPs (**left**) and EGCG–TiO_2_ NPs (**right**). Arrows indicate the value of the bandgap energy estimated by linear fit.

In order to determine the photocatalytic activity of both types of NPs, we estimated their optical bandgaps according to the Tauc plot method. The UV spectra were converted using the following equation:

(αhν)^2^ = k(hν − E_g_), (1)

where α is the absorption coefficient, hν represents the photon energy, E_g_ is the bandgap energy, and k is a constant. The bandgap energy theoretically calculated by linear fit of the Tauc plot was 2.71 eV for chem-TiO_2_ NPs and slightly higher (2.99 eV) for the green synthesized TiO_2_ particles (Figure 2). These values were comparable with the bandgap of standard TiO_2_ NPs (around 3.0 to 3.2 eV). The difference in bandgap between the two samples suggests that EGCG caused potential alteration in the photoactivity of the synthesized NPs.

### 3.2. Determination of the Functional Groups on the Surface of Green Synthesised TiO_2_ NPs

**Figure 3:**
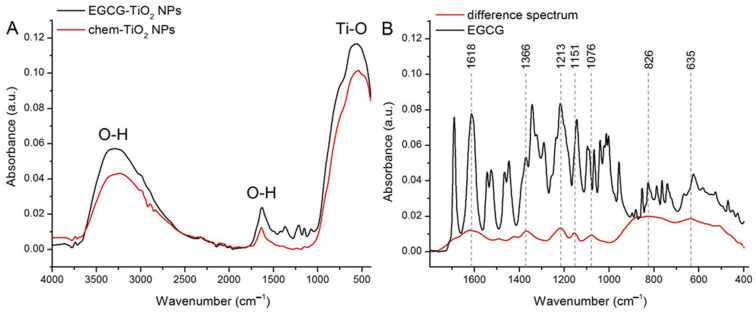
(**A**) FTIR spectra of chem-TiO_2_ NPs and EGCG–TiO_2_ NPs. (**B**) The result of spectra subtraction performed on chem-TiO_2_ NPs and EGCG–TiO_2_ NPs with a scaling factor of 1 (difference spectrum) and the FTIR spectrum of pure EGCG.

FTIR spectra of chem-TiO_2_ NPs and EGCG–TiO_2_ NPs were measured in the 400–4000 cm^−1^ range (Figure 3A,B). The spectra of both types of NPs present a large absorption band between 1000 and 400 cm^−1^ that comprises contributions from Ti-O bonds stretching [37]. The peaks corresponding to these vibrations are seen at 540 cm^−1^ (chem-TiO_2_ NPs) and 552 cm^−1^ (EGCG–TiO_2_ NPs), confirming the successful synthesis of NPs.

Both NPs present broad absorption bands with peaks at 3229 cm^−1^ (chem-TiO_2_ NPs) and 3317 cm^−1^ (EGCG–TiO_2_ NPs) that can be assigned to O-H stretching modes of surface hydroxyl groups from moist in the sample [38]. Similarly, the peaks at 1636 cm^−1^ (chem-TiO_2_ NPs) and ~1630 cm^−1^ (EGCG–TiO_2_ NPs) are associated with the bending of OH groups from water at the surface of NPs [38].

The spectrum of EGCG–TiO_2_ NPs presents some absorption bands between 1040 and 1520 cm^−1^ that have no correspondence in the spectrum of chem-TiO_2_ NPs. We expect these peaks to reflect the contribution of moieties originating from the EGCG solution. Therefore, we subtracted the spectrum of chem-TiO_2_ from that of EGCG–TiO_2_ and compared the result with the spectrum measured on pure EGCG powder (Figure 3B). The subtracted spectrum presents clear absorption bands centered at 1618 cm^−1^, 1366 cm^−1^, 1213 cm^−1^, 1151 cm^−1^, 1076 cm^−1^, 826 cm^−1^, and 635 cm^−1^. These absorption bands can be assigned to vibrations in aromatic C=C bonds (1618 cm^−1^), alcohol C-O bonds (1366 cm^−1^), aromatic O-H bonds (1213 cm^−1^), alcohol C-OH bonds (1151 cm^−1^) [39], polyphenols C-O-C bonds (1079 cm^−1^), and alkene C-H bonds (826 and 635 cm^−1^) [40]. These bands can also be noticed in the spectrum of pure EGCG, confirming the capping of TiO_2_ NPs with EGCG molecules in EGCG–TiO_2_ NPs.

### 3.3. SEM Analysis of Green Synthesised TiO_2_ NPs

**Figure 4:**
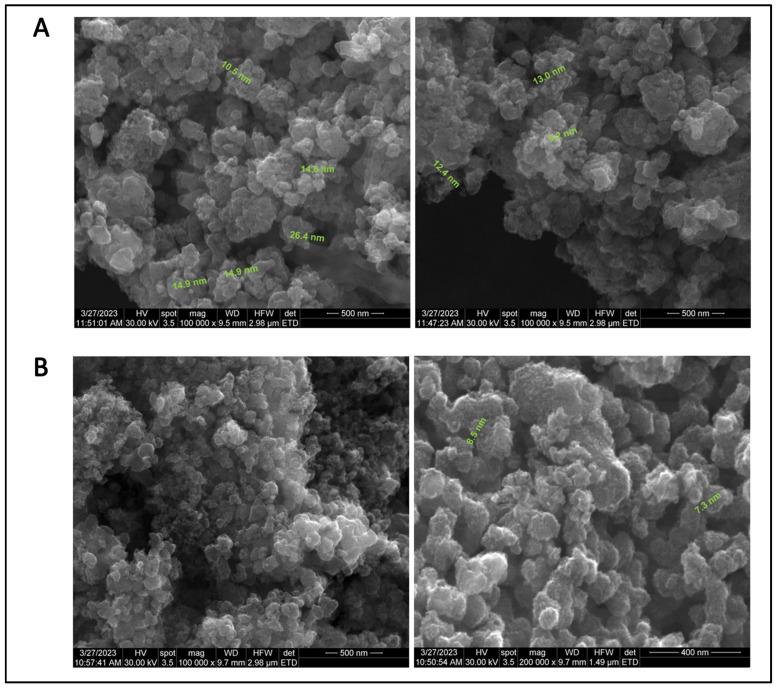
SEM micrographs of (**A**) chem-TiO_2_ NPs (scale bar: 500 nm) and (**B**) EGCG–TiO_2_ NPs (scale bar: 500 and 400 nm).

The analysis of SEM images provided information regarding the surface morphology, shape, and size of the particles. SEM micrographs revealed that the majority of synthesized particles were spherical, with nanometer dimensions and uniformly dispersed, but densely aggregated into clusters. The size of individual chem-TiO_2_ NPs observed by SEM was between 2.9 nm and 26.4 nm (Figure 4A). EGCG–TiO_2_ NPs appeared smaller, with a narrower size range, i.e., from 5.1 nm to 8.5 nm (Figure 4B).

### 3.4. Crystallographic Structure of Green Synthesised TiO_2_ NPs

**Figure 5:**
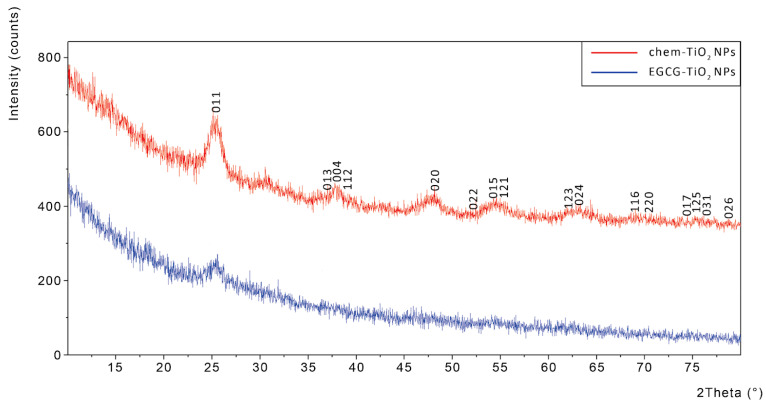
XRD spectra of chem-TiO_2_ NPs (red) showing Miller indices and EGCG–TiO_2_ NPs (blue), respectively.

The XRD analysis showed that chemically synthesized TiO_2_ NPs had a well-defined crystalline structure consisting of 73.3% anatase and 26.7% brookite as determined by Rietveld method. The XRD pattern of chem-TiO_2_ NPs showed that the major peaks were placed near the following 2θ angles: 25.4992° (corresponding to the (011) reflection of anatase), 37.9178° (corresponding to the (013), (004), (112) reflection planes of anatase), 47.7277° (corresponding to the (020) reflection of anatase), and 54.8805° (corresponding to the (015) and (121) reflection planes of anatase), 63.4528° (corresponding to the (123) and (024) reflection planes of anatase) (Figure 5).

In contrast with the data obtained for chem-TiO_2_ NPs, XRD spectrum revealed that the green synthesized NPs have not acquired a distinct crystalline structure. The most pronounced and visible peak corresponded to the (011) reflection plane, but the entire XRD spectrum suggested an amorphous nature for EGCG–TiO_2_ NPs. The increased amorphous nature of titania when EGCG is used in green synthesis can be attributed to the specific interactions between EGCG and TiO_2_ during the synthesis process. First of all, EGCG often serves as a stabilizing or capping agent during nanoparticle synthesis. The interaction between EGCG molecules and the surface of TiO_2_ NPs can lead to the formation of a protective layer around the NPs. This layer may inhibit the growth of well-defined crystal structures, favoring the formation of amorphous or less-crystalline phases. Secondly, EGCG contains multiple functional groups, including hydroxyl and catechol groups, which can form coordination complexes with metal ions. These chelation effects can influence the nucleation and growth of titania NPs, potentially leading to the formation of amorphous or less-crystalline structures. Also, the kinetics of the reaction, influenced by the presence of EGCG, can play a role in the final crystallinity of the titania NPs. EGCG may slow down the nucleation and growth processes, favoring the formation of amorphous structures due to the controlled and gradual development of NPs.

### 3.5. Hydrodynamic Diameter and Zeta-Potential of Green Synthesised TiO_2_ NPs

**Figure 6:**
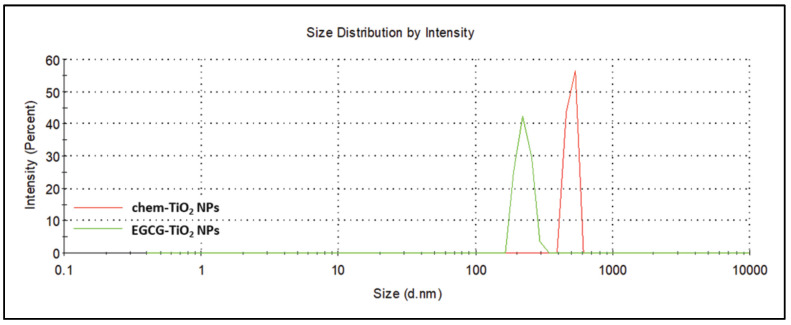
Representative DLS spectrum showing the particle size distribution of chem-TiO_2_ (red) and EGCG–TiO_2_ NPs (green).

The hydrodynamic diameters of the chemical and green synthesized TiO_2_ NPs were precisely measured by DLS (Figure 6). As shown in Table 1 and in accordance with SEM micrographs, the intensity-weighted hydrodynamic average diameter (Z-average) revealed a tendency to aggregation for both samples, but this was more pronounced in the case of chem-TiO_2_ NPs. Z-average of chem-TiO_2_ NPs exceeded 1000 nm while the green synthesized TiO_2_ NPs had a more reduced hydrodynamic diameter. To be precise, chem-TiO_2_ NPs dispersed in water showed an average size of 1469 ± 101.8 nm and EGCG–TiO_2_ NPs of 438.5 ± 22.56 nm, respectively. Accordingly, the polydispersity index of chemically synthesized TiO_2_ NPs had a higher value than that of the green synthesized ones.

When larger particles or agglomerates are present, as shown in SEM images, the scattered light signals obtained during DLS analysis may reflect the movement of these larger entities rather than individual particles. As a result, the measured hydrodynamic diameter tends to be overestimated, and the polydispersity index may increase, indicating a broader size distribution, as shown for chem-TiO_2_ NPs in Table 1.

Understanding the impact of large aggregates on DLS results is crucial for accurately interpreting the NP size and distribution in the sample. Additionally, discussing how the surface chemistry influences the formation of these aggregates provides insights into the complex interactions that affect the colloidal stability of NPs in the solution. One critical aspect that influences the aggregates’ formation is the surface chemistry of the NPs. TiO_2_ NPs typically exhibit surface charges that can be influenced by the pH of the solution. When the surface charge is insufficient to overcome attractive forces, such as van der Waals forces, nanoparticles may aggregate, leading to the formation of large chunks. The electrostatic repulsion provided by surface charges is crucial in preventing aggregation, and any deviation from optimal conditions can result in diminished stability. Functional groups present on the surface of NPs can interact with each other or with components in the solution, affecting their dispersion. Certain functional groups may promote agglomeration or aggregation, especially if they facilitate attractive forces between particles. Conversely, surface modifications that enhance repulsive interactions contribute to improved stability.

Furthermore, the zeta potential (Table 1) was measured, being crucial in understanding the stability of NPs in a solution. The electrostatic repulsion counteracts the attractive van der Waals forces that tend to bring particles together, thereby maintaining dispersion and stability in the colloidal system. Therefore, a higher absolute value of zeta potential (whether more negative or positive) signifies a stronger electrostatic repulsion between particles, which hinders aggregation and contributes to the overall stability of nanoparticles in the solution. The zeta potential measurements revealed that the synthesized particles were both negatively charged, but their stability was different. EGCG–TiO_2_ NPs were found to be very stable as their zeta potential had an average value of −56.5 ± 6.43 mV, indicating that the TiO_2_ NPs are surrounded by the negatively charged EGCG. The zeta potential average of the colloidal solution of chem-TiO_2_ NPs was higher than −25 mV. According to this result, the last-mentioned type of NPs might be considered on the limit of a favorable stability.

## 4. Discussion

In the present work, we proposed the synthesis of TiO_2_ NPs by hydrolysis of TTIP and further condensation of the reaction products in the presence of a pure EGCG solution. The reactions could be represented as follows:

Ti(C_3_H_7_O)_4_ + 4H_2_O → Ti(OH)_4_ + 4C_3_H_8_O (hydrolysis) (2)

2[Ti(OH)_4_] → Ti_2_O_7_H_6_ + H_2_O (condensation I) (3)

Ti_2_O_7_H_6_ → 2TiO_2_ + 3H_2_O (condensation II) (4)

Following particle cleaning and drying, approximately 0.6 g of NPs were produced relative to a total reaction mixture volume of 108 mL.

EGCG does not have an essential role in the formation of TiO_2_ NPs, as the mechanism that lies behind is described by the chemical reactions presented above and proved by our direct hydrolysis experiment. However, we consider the main advantage of using EGCG over other biomolecules for TiO_2_ NPs synthesis could come from the high binding affinity this molecule has for the titanium atoms exposed at the surface of particles. The strong interaction of polyphenols with TiO_2_ NPs was explained by the vicinal trihydroxy group within their structure. Li et al. [41] proved that the two vicinal trihydroxy groups that EGCG possesses allow its absorption on the surface of TiO_2_ NPs in high amounts even at low concentrations. Their results regarding the interaction of TiO_2_ NPs with polyphenols proved that EGCG–TiO_2_ complex had the strongest charge transfer interaction [41], described by the transferring of the biomolecule’s electrons to the conduction band specific to TiO_2_ [42]. This structural particularity of EGCG was actually the main reason we chose it for assisting the synthesis of TiO_2_ NPs. Further, this complex interaction might be the basis of the high stability of TiO_2_ NPs proved by us through measuring zeta potential. The abovementioned cited analysis of interaction was performed on the surface of already structured NPs. Considering our XRD data, the binding of EGCG to Ti atoms might occur during condensation, thus EGCG intervenes in the 3D structure formed by the intermediary products.

**Figure 7:**
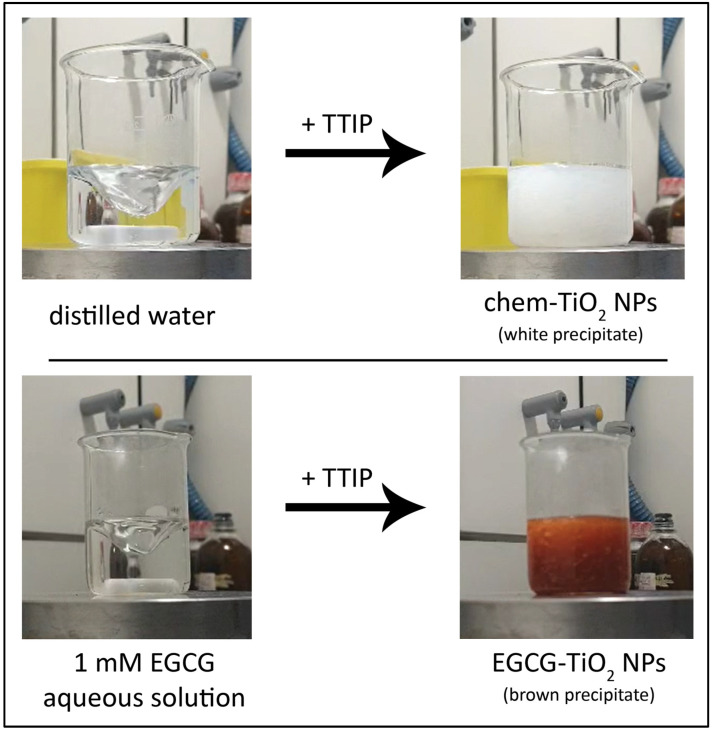
The color change of the solution when TTIP is added into the reaction mixture under stirring: chem-TiO_2_ sample (**top**), EGCG–TiO_2_ sample (**bottom**).

We observed that, upon the immediate dropwise addition of TTIP into water, the color of the mixture changed from transparent to white, while some precipitated particles have been formed. Similarly, the aqueous solution of EGCG turned from transparent to brown when TTIP was added (Figure 7). The visual observation of a color change into the reaction mixture has commonly been considered a first proof of the formation of NPs in studies dealing with green approaches [43,44,45].

The formation of TiO_2_ particles was further confirmed by UV-VIS spectroscopy. Spectra of the dried particles revealed an absorption peak at around 395 nm for both samples, which according to previous studies could be assigned to TiO_2_. Even though the specific absorbance peak slightly varied in studies dealing with green synthesis of TiO_2_ NPs, their spectrum was defined by a strong absorbance of the UV light, below the 400 nm wavelength [36,46,47,48]. The additional peaks occurring at 226 nm/232 nm might indicate residual traces from the TTIP precursor solution which registered various peaks up to the wavelength of 233 nm. Additional peaks, including those occurring at 276 nm/278 nm, might be explained also by intermediary products. The amplitude of these additional peaks was higher for EGCG–TiO_2_ NPs probably due to the overlapping with the UV absorbance of the catechin from their surface. According to our results, EGCG presented a maximum absorption at 274 nm. Previously, it was noticed that this was slightly red-shifted in relation to green tea whose peak occurred at 271.5 nm probably due to the presence of other catechins [49].

TiO_2_ is a semiconductor material with photocatalytic activity that has a bandgap value around 3.2 eV [50]. Its photoactivation occurs after exposure to UV radiation due to this broad difference between the valence and conduction bands. Most studies using plant extracts for the synthesis of TiO_2_ NPs reported particles with similar bandgap compared to bulk TiO_2_ [51,52,53]. In accordance with them, we estimated the bandgap of the chemically synthesized TiO_2_ NPs to be 2.71 eV. The bandgap of the green synthesized TiO_2_ NPs was higher, approaching 3 eV. These results revealed that the TiO_2_ NPs synthesized by us might be photocatalytically active. The difference between bandgap values of these samples might be explained by their crystal phase; chem-TiO_2_ NPs had a mixed anatase-brookite crystal phase that was shown previously to have a superior photocatalytic activity than anatase itself or commercial P25 Degussa (anatase–rutile mixture) [54]. The physical process was explained by Murakami et al. [55] through the reduced recombination of photogenerated holes and electrons in powders of TiO_2_ with mixed phases.

The presence of functional groups on the surface of green synthesized TiO_2_ NPs was revealed by the FTIR spectrum in which a distinct region between the wavenumbers 1800 and 400 cm^−1^ could be observed. That region is defined by a bunch of peaks that could be found previously in the infrared spectrum of green tea [56] and EGCG [57]. A similar infrared spectrum pattern was found previously in other types of metallic and metallic oxide NPs produced using green tea extract, including gold [20], silver [58], and copper oxide [26]. The presence of EGCG moieties at the surface of the TiO_2_ NPs should offer them improved properties, such as adhesiveness and biocompatibility [59]. Such polyphenol-containing NPs present important biomedical applications, including bioimaging, diagnosis and treatment of cancer or other diseases [59]. In particular, TiO_2_ NPs capped with natural compounds from plant extracts were reported to present biomedical applications based on their antibacterial and antifungal properties. Also, these posses photocatalytic applications and environmental applications involving wastewater treatment and heavy metal decontamination [60].

The spherical morphology and nanoscale dimensions of the synthesized TiO_2_ NPs were confirmed by SEM analysis. NPs are theoretically limited to 100 nm [61], while our samples were far below this value. In order to reveal the size range of different samples, randomly selected NPs from SEM images were measured. Our results showed that the size of chem-TiO_2_ NPs could be comparable with that of the commercial P25 Degussa NPs [62]. The smaller dimensions of EGCG–TiO_2_ NPs might be attributable to the capping effect of the polyphenol that prevented excessive polymerization of the alkoxide, hence limiting particles’ growth. This chemical mechanism is plausible considering that presence of EGCG into the reaction mixture was the only variable applied to the synthesis method. Previously, different studies revealed the capping efficiency of biomolecules [33].

The addition of EGCG into the reaction medium significantly influenced the formation of the crystalline structure of TiO_2_ NPs. The pattern of XRD revealed that green synthesized TiO_2_ NPs were amorphous in comparison with the chemically synthesized ones that had a major anatase component. The anatase crystalline phase is normally found at low calcination temperatures up to 600 °C, where its irreversible conversion into rutile takes place [63]. The amorphous nature of EGCG–TiO_2_ NPs might be explained by the intercalation of catechins into the 3D structure formed by the intermediary products during condensation. In contrast with our results, other studies generated anatase or rutile TiO_2_ NPs by green approaches depending on the calcination temperature applied [53,64]. However, our results were in accordance with those of Sankar et al. [65] that produced amorphous TiO_2_ NPs by using TTIP and *Azadirachta indica* aqueous leaf extract. The calcination step was not included in their method. In contrast, Nasrollahzadeh et al. [66] obtained rutile TiO_2_ NPs by using TiO(OH)_2_ and *Euphorbia heteradena Jaub* root extract also without calcination. For this reason, we tend to believe that the crystalline structure is influenced more by the nature of the phytocompounds used and less by the drying temperature of the NPs.

The EGCG molecules within the structure of TiO_2_ NPs had a beneficial effect on their stability. In general, NPs are considered stable when the zeta potential is under −25 mV or over +25 mV [67]. Chem-TiO_2_ NPs suspensions were at the limit of stability. The zeta potential of EGCG–TiO_2_ NPs significantly differed in comparison to the other type of TiO_2_ NPs and revealed that they present a total negative charge and generate suspensions with high stability. DLS analysis showed that the aggregation tendency of green synthesized TiO_2_ NPs was significantly reduced. The layer of functional likely groups onto the surface of NPs prevented the contact between particles. Our results were in accordance with other studies dealing with green synthesis of NPs using EGCG or green tea. For example, Chavva et al. [68] produced EGCG mediated gold NPs with a zeta potential of −72.57 mV. Similarly, silver NPs synthesized using green tea in optimized conditions had a zeta potential value of −62.6 mV [69].

## 5. Conclusions and Future Perspectives

Over the past years, several discoveries regarding the molecular processes of green chemistry involved in nanotechnology have been made. Therefore, there are different theoretical models that were developed to explain the mechanism responsible for the synthesis of green NPs. Some studies claimed that the formation of TiO_2_ NPs from TTIP is driven by the reducing power of the phytocompounds used during synthesis [70,71]. Our results revealed that EGCG had no essential role in the formation of TiO_2_ NPs, as their synthesis was determined by the lysis effect of water molecules on TTIP. However, we proved that EGCG could change the physicochemical properties of the obtained TiO_2_ NPs.

Working with pure EGCG allowed for better control and standardization of experiments, allowing us to study the effects of this particular compound without the interference from other components present in green tea extract. The main advantage of using EGCG in the synthesis of TiO_2_ NPs was a considerable improvement of particle stability. Furthermore, this compound influenced the size of NPs. On the other hand, EGCG probably had a negative impact on the crystalline structure of NPs, preventing the formation of anatase at a low drying temperature. The mathematical modelling of the bandgap energy suggested that EGCG decreased the conductivity of NPs, but both samples were estimated to be photocatalytically active. Grace to this property, the NPs obtained within this study could find applications in areas such as wastewater treatment and dye degradation.

Even though TiO_2_ NPs are commonly used for their photocatalytic property, EGCG–TiO_2_ NPs synthesized by us could be useful in other types of applications such as drug delivery. Considering that EGCG is a medicinal compound that degrades relatively easily, its incorporation into inorganic NPs might extend the lifetime of its bioactivity hence the possibility for a prolonged antioxidant or antifungal effect. Moreover, EGCG–TiO_2_ NPs offer advantages, being safer alternatives to classical TiO_2_ NPs that have cytotoxic activity manifested via reactive oxygen species generation during exposure to UV light. These are research directions that future studies dealing with green synthesis of TiO_2_ NPs could address and might contribute to the scaling up of green nano-synthesis as a viable alternative to the conventional one.

## Figures and Tables

**Table 1:** DLS and zeta potential measurements of chem-TiO_2_ and EGCG–TiO_2_ NPs suspensions in water.

Sample	Z-Average (d. nm)	Size Peak (d. nm)	Polydispersity Index (PdI)	Zeta Potential (mV)
chem-TiO_2_ NPs	1469 ± 101.8	589.9 ± 211.6	0.747 ± 0.271	−20.3 ± 1.27
EGCG–TiO_2_ NPs	438.5 ± 22.56	245.5 ± 18.28	0.469 ± 0.027	−56.5 ± 6.43

## Data Availability

Data are available on request from the corresponding author.
